# Management Strategies for Comorbid Supine Hypertension in Patients with Neurogenic Orthostatic Hypotension

**DOI:** 10.1007/s11910-021-01104-3

**Published:** 2021-03-09

**Authors:** Stuart H. Isaacson, Khashayar Dashtipour, Ali A. Mehdirad, Amanda C. Peltier

**Affiliations:** 1 Parkinson’s Disease and Movement Disorders Center of Boca Raton, 951 NW 13th Street, Bldg. 5-E, Boca Raton, FL USA; 2 Division of Movement Disorders, Department of Neurology, Loma Linda University School of Medicine, Loma Linda, CA USA; 3 Wright State University, Dayton VA Medical Center, Dayton, OH USA; 4 Department of Neurology and Medicine, Vanderbilt University, Nashville, TN USA

**Keywords:** Autonomic dysfunction, Blood pressure dysregulation, Droxidopa, Fludrocortisone, Midodrine, Pyridostigmine, Variable blood pressure

## Abstract

**Purpose of Review:**

In autonomic failure, neurogenic orthostatic hypotension (nOH) and neurogenic supine hypertension (nSH) are interrelated conditions characterized by postural blood pressure (BP) dysregulation. nOH results in a sustained BP drop upon standing, which can lead to symptoms that include lightheadedness, orthostatic dizziness, presyncope, and syncope. nSH is characterized by elevated BP when supine and, although often asymptomatic, may increase long-term cardiovascular and cerebrovascular risk. This article reviews the pathophysiology and clinical characteristics of nOH and nSH, and describes the management of patients with both nOH and nSH.

**Recent Findings:**

Pressor medications required to treat the symptoms of nOH also increase the risk of nSH. Because nOH and nSH are hemodynamically opposed, therapies to treat one condition may exacerbate the other. The management of patients with nOH who also have nSH can be challenging and requires an individualized approach to balance the short- and long-term risks associated with these conditions.

**Summary:**

Approaches to manage neurogenic BP dysregulation include nonpharmacologic approaches and pharmacologic treatments. A stepwise treatment approach is presented to help guide neurologists in managing patients with both nOH and nSH.

## Introduction

The development of hemodynamic abnormalities often results as a consequence of the autonomic failure that occurs in patients with Parkinson disease (PD) and other neurodegenerative α-synucleinopathies (e.g., multiple system atrophy [MSA], pure autonomic failure) [1••]. One manifestation is neurogenic orthostatic hypotension (nOH), a sustained blood pressure (BP) drop upon standing. nOH is caused by the inability of the autonomic nervous system to maintain BP in response to standing due to baroreflex failure causing inadequate norepinephrine release with impaired vasoconstriction of peripheral and splanchnic vessels [2, 3]. Up to 70% of patients with nOH may also have neurogenic supine hypertension (nSH) [4, 5], which differs from essential hypertension in that individuals with nSH have increased BP when supine, may be normotensive when seated, and are frequently hypotensive when standing [1••, 6••].

Both nOH and nSH are associated with significant morbidity. Symptoms of nOH can include lightheadedness, orthostatic dizziness, cognitive slowing, sleepiness, presyncope, and syncope [7, 8] and may lead to an increased risk of falls, cognitive impairment, social withdrawal, and exercise intolerance [9–11]. nSH is commonly asymptomatic [6••]; however, it may have potential long-term cardiovascular risks including, but not limited to, left ventricular hypertrophy and stroke [12–14]. When managing patients with symptomatic nOH, clinicians should evaluate supine BP before and during treatment. Although concomitant management of symptomatic nOH and nSH can be inherently challenging because they are hemodynamically opposite forms of BP dysregulation and attempts to improve one condition may exacerbate the other, it is necessary for clinicians to address coexisting nOH and nSH in patients. This review considers autonomic failure pathophysiology, clinical features, BP evaluation (e.g., circadian BP variability, postural change, meals), nonpharmacologic approaches, and pharmacologic treatment strategies for the management of patients with coexisting nOH and nSH.

## Pathophysiology of Blood Pressure Control Abnormalities Associated with Autonomic Nervous System Dysfunction

Both nOH and nSH occur because of autonomic nervous system (ANS) failure and the consequent inability of the cardiovascular system to adequately respond to postural change [6••, 15]. When assuming the standing position, a large blood volume shifts to the splanchnic and lower-extremity vasculature [15]. To maintain adequate perfusion of the brain and other organs, baroreceptors in the aortic arch and carotid sinus detect these changes and signal for vasoconstriction and increases in heart rate and cardiac output through a pathway involving both the central and peripheral branches of the ANS [3, 15]. The signaling for blood vessel constriction required to maintain BP in response to standing is mediated by norepinephrine release from postganglionic sympathetic nerves [3, 16]. Attenuated increases in plasma norepinephrine that occur from supine to standing positions have been demonstrated in patients with PD, MSA, and pure autonomic failure [17], and these inadequate orthostatic increases in plasma norepinephrine levels underlie the occurrence of nOH in these conditions [18]. nOH can be caused by central or peripheral degeneration and disruption of the ANS [18–20]. In PD and pure autonomic failure, there is both central (e.g., locus coeruleus) and peripheral neurodegeneration (i.e., postganglionic sympathetic denervation), whereas in MSA, the ANS defects are primarily central, and postganglionic sympathetic nerves remain intact [18, 21]. nOH can also be caused by other peripheral neuropathies that result in impaired autonomic reflexes (e.g., diabetes, autoimmune disorders, chemical/toxin induced) [22, 23].

Dysfunction within the ANS pathways for BP homeostasis causes an overall inability to regulate BP in response to postural change, resulting in the coexistence of nOH and nSH. The specific mechanisms contributing to nSH may differ in patients depending on neurodegeneration pathophysiology (i.e., central and/or peripheral). In patients with intact postganglionic sympathetic innervation (e.g., MSA), nSH may result when impaired baroreflex function cannot adequately compensate for norepinephrine released by the intact postganglionic sympathetic nerves [6••, 24]. However, in patients with postganglionic sympathetic denervation and low norepinephrine plasma levels (e.g., pure autonomic failure, PD), nSH may be caused by dysfunction within the baroreflex-mediated BP control pathways and hypersensitivity to residual norepinephrine release [6••, 24]. Other abnormalities in maintaining physiologic control of BP also likely contribute to the development of nSH. Arnold et al. demonstrated that plasma angiotensin II levels are elevated in autonomic failure [25], suggesting that nOH may lead to chronic activation of the renin-angiotensin system, which could contribute to the development of nSH [15, 26]. Patients with autonomic failure experience increased nocturnal sodium excretion and decreased daytime sodium excretion, and many have renal impairment that may also be related to nSH [14, 27]. The development of nSH may also involve other disruptions in the baroreflex including the vasomotor center and effector organs (e.g., heart, arterioles, veins) [28, 29], as well as increases in central blood volume due to the shifting of fluid away from the periphery.

More research is needed to better understand the distinct pathophysiology and clinical effects of BP dysregulation in autonomic failure disorders. For example, although it is known that there is poor prognosis in patients with nOH or nSH, it is unknown whether the poor prognosis is due to underlying autonomic failure, BP variability, chronic hypoperfusion, vasculature changes, or a combination of these [6••, 30]. A better understanding of the specific pathophysiology in patients with autonomic failure may result in better approaches to mitigate the long-term clinical impact of autonomic failure.

## Clinical Features and Consequences of Blood Pressure Dysregulation in Patients with Autonomic Dysfunction

### Neurogenic Orthostatic Hypotension

The estimated prevalence of nOH in autonomic failure disorders varies by underlying diagnosis, ranging from 30 to 40% of patients with PD, > 75% of patients with MSA, and 100% of patients with pure autonomic failure [31–34]. Orthostatic hypotension (OH) is defined as a reduction in systolic BP of ≥ 20 mmHg or diastolic BP of ≥ 10 mmHg within 3 min of standing (or ≥ 60° head-up tilt using a tilt-table) [2]; nOH is defined as OH caused by autonomic dysfunction [26]. Clinically, nOH can be distinguished from other causes of OH, such as use of certain medications and hypovolemia and/or by an inadequate cardioacceleratory response (< 15 bpm elevation of heart rate) upon standing [26]. Patients with nOH can be asymptomatic despite a substantial drop in orthostatic BP if standing BP remains within the range of cerebral perfusion pressure and cerebral autoregulation. When standing BP falls below the cerebral autoregulation threshold, symptoms emerge as a result of cerebral hypoperfusion. The lower BP threshold of cerebral autoregulation varies among individuals, but one study of patients with nOH found that most patients had symptoms if mean upright BP was < 75 mmHg at heart level or standing systolic BP < 100 mmHg [8]. For this reason, symptomatic nOH is most reflective of a standing systolic BP below that of an individual patient’s lower range of cerebral autoregulation and not the magnitude of the orthostatic drop in BP [8].

Common symptoms of nOH include lightheadedness, orthostatic dizziness, presyncope, and syncope, but patients may also report cognitive impairment, headache, impaired vision, fatigue, general weakness, shortness of breath while standing, neck and shoulder pain, angina, and falls [3, 26, 31, 35]. nOH symptoms can adversely affect patients’ daily function and quality of life. In a survey of 363 patients with nOH, most respondents (87%) indicated that nOH symptoms affected their daily activities, such as physical activities/exercise, housework, traveling, and time spent out of the house [9]. Patients also felt that nOH symptoms decreased their quality of life (59%), caused a loss of independence (42%), and drastically changed their lives (40%) [9]. In addition to the functional and psychosocial burden associated with symptomatic nOH, risk of fainting, falls, and related injuries is a major concern. Data from nOH patient cohorts indicate that falls are a frequent occurrence and present a safety threat because of injury potential [9, 36–38]. Patients with PD and nOH have greater fall-related and all-cause healthcare utilization (e.g., emergency department visits, hospitalizations) and greater healthcare costs (approximately 50–250% more in adjusted costs) compared with patients with PD without nOH [39, 40]. nOH has also been associated with increased mortality in patients with PD [41].

### Neurogenic Supine Hypertension

In patients with autonomic failure, the estimated prevalence of nSH varies depending on the underlying cause of autonomic failure and how nSH is defined, with estimates ranging from 21 to 46% of patients with PD [8, 42, 43], up to 50% of patients with MSA, and up to 70% of patients with pure autonomic failure [5, 42, 44]. Supine hypertension is defined as a systolic BP ≥ 140 mmHg and/or diastolic BP ≥ 90 mmHg after ≥ 5 min of supine rest and can be classified as mild (140–159/90–99 mmHg), moderate (160–179/100–109 mmHg), or severe (≥ 180/≥ 110 mmHg) [1••, 6••]. Supine BP is typically measured with patients in a 30° elevated recumbent posture, but has also been reported with patients fully supine. Although nSH is usually asymptomatic, some patients may experience nonspecific symptoms such as dizziness, fatigue, headache, and blurred vision [6••, 28]. Additionally, nSH is associated with nocturnal pressure natriuresis with frequent nocturia leading to considerable sleep disruption. Nocturia also results in volume depletion overnight, exacerbating nOH symptoms in the morning hours [6••].

The pathophysiology of nSH is different than that of essential hypertension. There is limited understanding of whether the extreme but episodic BP variability in patients with autonomic failure (i.e., high BP when supine, low BP when standing) has similar morbidity risk as seen in essential hypertension (e.g., systemic vasculature damage). nSH has been associated with left ventricular hypertrophy, as well as an increased risk for end-target organ damage and premature death [12]. Nighttime systolic BP and 24-h BP (independent of BP variability) have been linked to the development of structural cardiac changes, such as increased left ventricular mass and left ventricular hypertrophy, in patients with autonomic failure compared with normotensive individuals [45]; the extent of these cardiac structural changes was similar to that observed in hypertensive patients [13, 45, 46]. nSH may also contribute to renal impairment. Significantly higher serum creatinine levels and lower estimated glomerular filtration rates were found in patients with pure autonomic failure and nSH compared with healthy controls [27].

### Altered Diurnal Blood Pressure Patterns

Another clinical feature in patients with autonomic failure is blunted nocturnal “dipping” of BP levels [6••]. A normal circadian BP pattern includes nocturnal dipping of ≥ 10% from daytime values; however, patients with nSH may have nocturnal BP decreases of < 10% (“reduced dippers”), have no nocturnal BP drop (“nondippers”), or elevated nocturnal BP (“reverse dippers”) [6••, 47]. Data suggest that abnormal circadian BP patterns may be observed in approximately two-thirds or more of patients with autonomic failure disorders [48–50]. There are limited data regarding the clinical effects of blunted nocturnal BP dipping specifically in patients with autonomic failure. In a study of patients with PD, left ventricular mass was significantly higher in patients with reverse dipping (defined as a change in day-night BP < 0%) compared with nonreverse dippers (*P* = 0.005); the increases in left ventricular mass observed in reverse-dipping patients with PD were similar to those observed in patients with essential hypertension [51•]. These findings of cardiovascular risk associated with blunted dipping in an autonomic failure population are consistent with data from general population studies that showed an association between greater morbidity and mortality (i.e., increased stroke risk, heart, vasculature, and kidney damage) with altered diurnal BP patterns [52–57].

## Management of neurogenic orthostatic hypotension when neurogenic supine hypertension is also present

When treating patients with nOH, the aim of management is to increase standing systolic BP into the range of cerebral autoregulation to improve symptoms and the ability to participate in daily activities, as well as to reduce the risk of falls [1••, 26, 58]. However, when significant nSH is also present, management attempts to raise standing BP will invariably raise supine BP. The likelihood and consequences of the short- and long-term risks posed by each condition, along with evaluation of comorbidities, overall life expectancy, and concurrent medications for the individual patient, should be continually assessed. Both nonpharmacologic approaches (e.g., review and adjust medications with hypotensive effects, increase fluids and salt intake, make postural changes in stages, use of compression garments) and pharmacologic pressor medications are useful in the treatment of symptomatic nOH [26, 59]. Often, effective improvement of nOH symptoms requires treatment with pressor agents (Table 1) [50, 60–83] to increase standing BP above the lower range of cerebral perfusion pressure and cerebral autoregulation [26, 59]. When treating the symptoms of nOH, consideration of the risk/benefit profile associated with individual treatments, particularly with regard to supine BP increases, is needed.

**Table 1:** Summary of safety and BP effects of common pharmacologic treatments for OH/nOH [50, 60–83]

Drug	Droxidopa	Midodrine	Fludrocortisone	Pyridostigmine
MOA	● NE prodrug ● Conversion to NE induces vasoconstriction and increases BP	● α1-Adrenoreceptor agonist prodrug ● Raises BP by increasing vascular resistance	● Raises BP by increasing intravascular volume via renal sodium reabsorption	● AChE inhibitor ● Enhances ganglionic transmission
Indication	● Treatment of orthostatic dizziness, lightheadedness, or the “feeling that you are about to black out” in adult patients with symptomatic nOH caused by primary autonomic failure (PD, MSA, and pure autonomic failure), DβHD, or NDAN	● Treatment of symptomatic OH	● Off-label use^a^	● Off-label use^b^
Dosage	● 100–600 mg TID (per prescribing information)	● 10 mg TID (per prescribing information)	● 0.1–0.2 mg/day; off-label use	● 30–60 mg; 1–3 times/day; off-label use
PD/PK data	● *t*_1/2_, 2.5 h ● *t*_max_, 2 h ● Peak pressor effect 3.5 h after dosing (coincident with NE, *t*_max_, 3–4 h)	● *t*_1/2_, 3–4 h ● *t*_max_, 1–2 h ● Peak effects: 1 h after dosing	● Plasma *t*_1/2_: ≥ 3.5 h; biological half-life: 18–36 h ● *t*_max_, 2 h ● Peak effects not reported for nOH/OH	● *t*_1/2_, 1.8 h ● t_max_, 1.7 h ● Peak effects not reported for nOH/OH
Key BP-related clinical findings				
Standing	● Mean SBP increase in RCTs, 12 mmHg ● Significant increase in daytime SBP from baseline in 24-h BP study (mean increase, 8.4 mmHg; *P* = 0.01)	● Mean SBP increase (2 meta-analyses), 17–21 mmHg	● Mean SBP increase vs baseline (1 study), 7 mmHg (0.2 mg)	● Mean SBP increase vs baseline (3 studies), 0–13 mmHg (60 mg)
Sitting	● Not available	● Mean/median SBP increase (2 RCTs), ~ 20–40 mmHg (5–10 mg)	● Mean SBP increase vs baseline (1 study), 16 mmHg (0.2 mg)	● Mean SBP increase vs baseline (1 study), 4 mmHg (60 mg)
Supine	● Mean BP ≤ 140 mmHg for 6 h after dosing (mean dose, 1147 mg) ● No significant increase in night-time SBP in 24-h BP study (mean increase, 7.8 mmHg^c^ *P* = 0.12)	● Mean SBP > 160 mmHg for up to 5 h after dosing (10 or 20 mg) ● Mean SBP increase (3 RCTs), 3–19 mmHg (2.5–10 mg)	● Mean SBP increase vs baseline (1 study), 15 mmHg (0.2 mg) ● Raises nocturnal (i.e., supine) BP to a greater magnitude than daytime (i.e., standing) BP (mean, 156 vs 134 mmHg)	Mean SBP increase vs baseline (4 studies), 2–11 mmHg (60 mg)
SH rates	● 1- to 2-wk RCTs^d^ o SBP > 160 mmHg: 9.9% (PBO, 6.1%) o SBP > 180 mmHg: 3.1% (PBO, 1.5%) o SBP > 200 mmHg: 0% (both groups) • 8- to 10-wk RCTs^d^ o SBP > 160 mmHg: 28.9% (PBO, 24.1%) o SBP > 180 mmHg: 7.9% (PBO, 4.6%) o SBP > 200 mmHg: 3.5% (PBO, 0.9%) ● Rate of night-time SBP elevation in 24-h BP study (vs off treatment) o SBP >160, 56% (off, 44%) o SBP >180, 17% (off, 28%) o SBP >200, 6% (off, 0%)	● Rates of supine SBP > 200 mmHg (single-dose study) o 17% (10 mg) o 41% (20 mg) ● 4 wk of active treatment (10 mg), 6% experienced SH (≥ 180/110 mmHg) leading to study discontinuation ● 4 wk of active treatment (2.5–10 mg TID), 8% experienced SH (≥180/110 mmHg); led to study discontinuation in 4/6 cases	● SH rates not available	● SH rates not available
SH-specific warning (in PI)	Monitor supine BP before and during treatment and more frequently when increasing doses. Elevate the head of the bed during rest or sleep; BP should be measured in this position. If SH persists, reduce or discontinue (boxed warning)	Can cause marked elevation of supine BP; should be used in patients whose lives are considerably impaired despite standard clinical care (boxed warning)	Dosage and salt intake should be carefully monitored to avoid the development of hypertension (warning)	None
Common AEs	● Headache ● Dizziness ● Nausea ● Hypertension	● Supine and seated hypertension ● Paresthesia and pruritus (scalp) ● Piloerection ● Chills ● Urinary urgency, retention, frequency	● Hypertension ● Edema ● Cardiac enlargement ● Congestive heart failure ● Potassium loss ● Hypokalemic alkalosis	● Nausea ● Vomiting ● Diarrhea ● Abdominal cramps ● Increased salivation ● Excessive sweating ● Urinary incontinence

*AChE* acetylcholinesterase, *AE* adverse event, *BP* blood pressure, *DβHD* dopamine β-hydroxylase deficiency, *MOA* mechanism of action, *MSA* multiple system atrophy, *NDAN* nondiabetic autonomic neuropathy, *NE* norepinephrine, *nOH* neurogenic orthostatic hypotension, *OH* orthostatic hypotension, *PBO* placebo, *PD* Parkinson disease, *PD/PK* pharmacodynamic/pharmacokinetic, *PI* Prescribing Information, *RCT* randomized controlled trial, *SBP* systolic blood pressure, *SH* supine hypertension, *t*_*1/2*_ half-life, *TID* three times daily, *t*_*max*_ = time to peak concentration

^a^Indicated as partial replacement therapy for primary and secondary adrenocortical insufficiency in Addison disease and for the treatment of salt-losing adrenogenital syndrome

^b^Useful in the treatment of myasthenia gravis

^c^Daily dose of droxidopa taken ≥ 4 h before bedtime

^d^Head and torso elevated 30°

### Nonpharmacologic Management of Neurogenic Orthostatic Hypotension

Nonpharmacologic management of nOH focuses on patient education, volume expansion, and mechanical increase of vascular return. Patients should be educated about standing slowly and avoiding heat, large meals, prolonged standing, the use of daytime antihypertensives, and dehydration, as well as about diurnal variations in BP and the lower BP present in morning hours [3, 26]. Volume expansion is encouraged with liquid hydration, liberal use of table salt or salt tablets, and 30° elevation of the head of the bed overnight to reduce nocturnal diuresis and improve morning BP [3, 26]. Mechanical compression of the lower extremities and abdomen with waist-high compression stockings and abdominal binders will increase blood return into the circulation [3, 26]. Additionally, patients can be educated to contract the muscles of the buttocks and legs when they stand [3]. Symptoms of nOH should be reassessed after two weeks of nonpharmacologic treatment for the need to add pharmacologic therapy.

### Pharmacologic Management of Neurogenic Orthostatic Hypotension

Pharmacologic management of nOH focuses on volume expansion and vasoconstriction. The most commonly used medications are the mineralocorticoid fludrocortisone, the acetylcholinesterase inhibitor pyridostigmine, the direct α_1_ receptor agonist midodrine, and the norepinephrine precursor droxidopa [26]. Other less commonly used medications to raise BP include central norepinephrine reuptake inhibitors and other medications that lead to BP increases via various mechanisms [15].

The mineralocorticoid fludrocortisone is used off-label for the treatment of OH [84] including nOH (Table 1). There are some clinical data to suggest that fludrocortisone is associated with BP increases in patients with nOH (due to PD or diabetic neuropathy); however, study design (i.e., case series) and small sample size considerations limit the overall assessment of effects [67, 85, 86]. BP elevations with fludrocortisone are initially observed 4 to 14 days after treatment initiation [87•, 88], but effects on nOH symptoms have not been rigorously evaluated. In patients with coexistent nOH and nSH, the volume expansion effects of fludrocortisone may limit its use because it raises supine BP to a greater magnitude than standing BP [68, 89]. During treatment, serum electrolytes should be regularly monitored, and pedal edema is a frequent dose-limiting side effect [90, 91].

Pyridostigmine, an acetylcholinesterase inhibitor, is used off-label to treat nOH because it enhances cholinergic neurotransmission at sympathetic ganglia. This increased cholinergic tone can result in increased norepinephrine release during orthostatic stress (Table 1) [26, 80, 82]. Because of this mechanism of action, it is thought that pyridostigmine can be used to treat nOH without exacerbating nSH [26, 80–82]. Studies of pyridostigmine treatment have shown increases in standing BP and improvements in orthostatic symptoms without significant effects on supine BP [80, 82], although these positive effects on nOH symptoms and standing and supine BP have not been consistently demonstrated in all trials [67, 81, 83]. Some of these differences may be due to the specific patient cohorts examined because the effects of pyridostigmine likely are related to residual sympathetic function [26, 81]. Potential side effects of pyridostigmine include nausea, vomiting, diarrhea, abdominal cramps, increased salivation, excessive sweating, and urinary incontinence [26, 78].

Midodrine is approved for the treatment of symptomatic OH (all causes). Approval was based on an increase in BP 1 min after standing (Table 1) [62], although clinical benefits (e.g., improved symptoms, ability to perform daily activities) were not confirmed in the registration trials [62]. The effectiveness of midodrine for increasing standing BP and improving OH symptoms is supported by findings of several subsequent randomized clinical trials [63–65]. Midodrine significantly increases standing systolic BP, but its use may be limited because of the magnitude of increases in sitting and supine BP (Table 1) [63, 64, 73–77]. In a single-dose study, midodrine treatment showed dose-dependent effects on incidence of nSH after 10- and 20-mg doses [65]. In other longer studies (4 weeks of active treatment), rates of study discontinuation due to nSH were 5 to 6% [63, 64]. Patients should be made aware that they should not be supine for 4 h after taking a dose of midodrine. Effective dosing can be limited by high BP when seated. Frequently reported side effects include scalp paresthesia/pruritus, piloerection, and urinary retention/urgency [62].

Droxidopa was approved by the US Food and Drug Administration for the treatment of symptomatic nOH [26]. It is the only agent approved based on symptomatic improvement, with a labeled indication for the treatment of orthostatic dizziness, lightheadedness, or the “feeling that you are about to black out” in adult patients with symptomatic nOH caused by primary autonomic failure (PD, MSA, and pure autonomic failure), dopamine β-hydroxylase deficiency, and nondiabetic autonomic neuropathy (Table 1) [60].

Clinical trials have demonstrated that droxidopa improves the symptoms of nOH and their effect on daily activities and is generally well tolerated with a low risk for the development of nSH [50, 61, 92, 93]. In clinical trials, rates of supine systolic BP > 160, > 180, and > 200 mmHg were not markedly different in patients receiving droxidopa versus placebo, although these elevated BP readings occurred more frequently in the droxidopa group (Table 1) [61]. Generally similar rates of nighttime BP readings > 160, > 180, and > 200 mmHg were found on and off droxidopa treatment in another small short-term study (*N* = 18; 4–5 weeks “on” then 24 h “off”), and the authors concluded that droxidopa had an “acceptably low” estimated 10 to 20% risk of increasing nocturnal nSH [50]. Although these data provide important information about the risks of nSH with droxidopa, interpretation may be limited for patients with severe preexisting hypertension (seated or supine BP ≥ 180/110 mmHg), who were excluded from the clinical trials of droxidopa [61].

### Comparisons of Fludrocortisone, Pyridostigmine, Midodrine, and Droxidopa

There are no direct comparison trials between fludrocortisone, midodrine, and droxidopa regarding efficacy, tolerability, or risk of nSH; pyridostigmine has been compared with fludrocortisone or midodrine in 2 small studies [67, 83]. A recent Bayesian mixed-treatment meta-analysis compared droxidopa and midodrine using clinical trial data. Both medications were associated with significant increases in standing systolic BP versus placebo (droxidopa, 6.2 mmHg [95% credible interval (CrI), 2.4–10.0]; midodrine, 17 mmHg [95% CrI, 11.4–23.0]) [76•]. This meta-analysis found that the relative risk of nSH for droxidopa was not significant (1.4 [95% CrI, 0.71–2.7] vs placebo), whereas there was a significant corresponding relative risk for midodrine (5.1 [95% CrI, 1.6–24.0] vs placebo) [76•]. To our knowledge, no similar nSH comparisons that include fludrocortisone have been reported. However, in a retrospective analysis of patients prescribed midodrine (*n* = 797) or fludrocortisone (*n* = 1324), the risk of all-cause hospitalization was greater for patients who received fludrocortisone versus midodrine (adjusted incidence-rate ratio [aIRR], 1.20; 95% confidence interval [CI], 1.02–1.40) and the hospitalization risk associated with fludrocortisone was more pronounced in patients with a history of congestive heart failure (aIRR vs midodrine, 1.42; 95% CI, 1.07–1.90) [94•].

In a crossover study of pyridostigmine and fludrocortisone in 13 patients with PD, increases in standing or supine BP from baseline were found for fludrocortisone but not pyridostigmine [67]. Neither drug improved nOH symptoms [67]. In a 3-month open-label study of pyridostigmine and midodrine, both pyridostigmine and midodrine ameliorated orthostatic BP reductions and improved nOH symptoms, but midodrine’s effects on symptoms were more pronounced [83]. In this study, significant increases in supine systolic BP (8–11 mmHg) were observed with pyridostigmine but not midodrine [83]. However, the generalizability of these findings is limited by the low doses of midodrine used (2.5–5.0 mg, 2 times daily) and the small sample sizes studied (29 patients per treatment arm).

## Stepwise Treatment Approach for Managing Supine Hypertension in Patients with Neurogenic Orthostatic Hypotension

**Fig 1:**
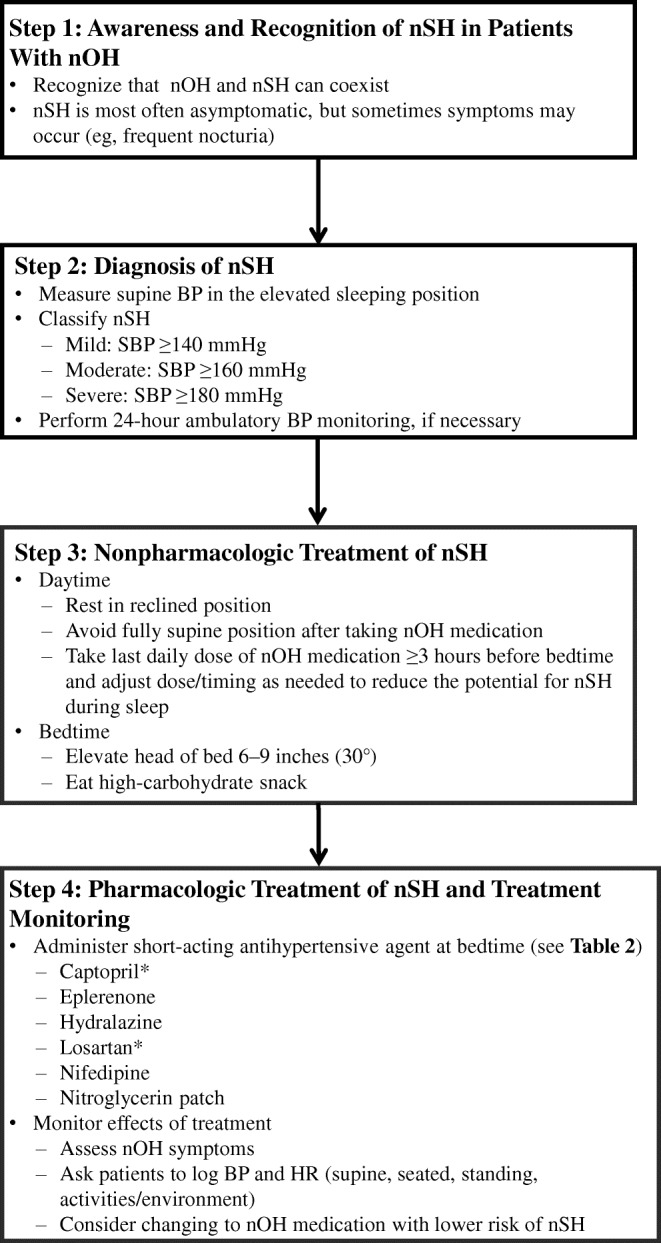
Stepwise treatment approach for managing nSH in patients with nOH [1••, 26, 59]. *BP* blood pressure; *HR* heart rate; *nOH* neurogenic orthostatic hypotension; *nSH* neurogenic supine hypertension; *SBP* systolic blood pressure. *Recommended based on the clinical experience of the authors

Because nSH can adversely affect patients’ morbidity risks, clinicians should identify patients with nOH who also have coexisting nSH to help optimize treatment strategies. However, because pressor agents used to treat symptoms of nOH can increase supine BP, and antihypertensive agents used to treat nSH can worsen the symptoms of nOH, an appropriate management strategy can be a clinical conundrum. A suggested treatment approach for evaluating and managing coexistent nOH and nSH based on current published evidence (clinical data and recommendations) and our clinical experience is shown in Fig. 1 [1, 26, 59].

### Step 1: Awareness and Recognition of Neurogenic Supine Hypertension in Patients With Neurogenic Orthostatic Hypotension

Although some patients with nSH may report frequent nocturia or other nonspecific symptoms (e.g., headache), most often, nSH is asymptomatic [6••]. Therefore, the fundamental way to identify nSH in patients with nOH is to measure BP in a “semi-recumbent” supine position (i.e., 30° horizontal elevation of the head) because patients at risk of nSH should typically avoid being in a fully supine position, including when sleeping.

### Step 2: Diagnosis of Neurogenic Supine Hypertension

The presence and magnitude of nSH can be evaluated clinically by measuring BP immediately after the patient assumes their semi-recumbent sleeping position and then after 5 min of rest in that position [6••, 26]. If required, BP assessments for nOH can also be conducted after the patient stands [26]. Because BP may fluctuate overnight [49], 24-h ambulatory BP monitoring can sometimes be useful to evaluate overnight BP and identify abnormal circadian patterns (e.g., reverse or reduced dipping) [6••, 26]. Daily BP patterns may also be assessed through a home BP log kept by the patient. Patients should be encouraged to record BP and heart rate at home (e.g., seated and standing before and after meals, in their semi-recumbent sleeping position at bedtime). These home BP logs can be particularly useful in the evaluation of nOH before implementing pharmacologic treatment for nSH and to evaluate effects after these treatments are initiated (Fig. 1, step 4).

### Step 3: Nonpharmacologic Treatment of Neurogenic Supine Hypertension

There are several nonpharmacologic strategies that can be used to reduce nSH (Fig. 1) [1••, 26, 59]; information about these should be provided as part of the patient’s education about nOH.

- Elevating the head of the bed can reduce supine BP [14, 95]. During nighttime, patients should sleep with the head of the bed elevated at least 6 to 9 inches (approximately 30°) [59]. If nSH persists, the angle of recumbence can be increasingly elevated, and some patients may need to sleep in a near-seated position (eg, in a recliner) to reduce supine hypertension [59]. A head-up sleeping position will reduce BP and natriuresis while keeping the renin-angiotensin system activated so that there will be less of a BP drop in the morning [1••, 26, 59, 96].
- To reduce nocturnal hypertension, patients can be advised to eat a high-carbohydrate snack at bedtime, which not only lowers BP by directing blood flow to the splanchnic circulation, but also causes insulin release (which has direct vasodilator properties) [97] with resultant vasodilation [1••, 98].
- During the daytime, patients using a pressor medication for nOH should avoid the supine position. To reduce the potential for nSH at night, the last daily dose of any nOH pressor medication should be taken at least 3 to 4 hours before bedtime [60, 62].
- Nonpharmacologic treatments for nSH should be continued even when pharmacotherapy is implemented.

### Step 4: Pharmacologic Treatment of Neurogenic Supine Hypertension and Treatment Monitoring

If nSH persists despite the use of nonpharmacologic measures, then treatment with a short-acting antihypertensive agent at bedtime may be necessary (Table 2) [1••, 25, 26, 44, 99–106]. Generally, pharmacologic management of nSH may be required for patients with severe nSH (systolic/diastolic BP ≥ 180/≥110 mmHg) and may be considered for patients with moderate nSH (BP 160–179/100–109 mmHg) based on individual patient circumstances and risk profile [6••, 26]. Short-acting agents are preferred to minimize chances that antihypertensive effects will persist into morning and worsen nOH symptoms when the patient arises for the day [1••]. Based on our clinical experience and previous published guidance, the following recommendations regarding agents and subsequent BP monitoring are provided.

- Administer a short-acting antihypertensive agent at bedtime or in the evening after dinner (Table 2) [1••, 26, 99–102, 105, 106].
- After therapy is initiated and at regular intervals, nOH symptoms and the patient’s home BP log should be re-evaluated (Fig. 1).
- If symptoms of nOH emerge or are exacerbated in the morning, consider adjusting the timing of the antihypertensive agent to earlier in the evening after dinner.

**Table 2:** Pharmacologic treatments for neurogenic supine hypertension [1••, 25, 26, 44, 99–106]

Medication (mechanism/class)	Typical dose	Half-life
Captopril (angiotensin-converting enzyme inhibitor) [26]	25 mg at bedtime	< 3 h [101]
Eplerenone (mineralocorticoid receptor antagonist) [25]	50 mg at bedtime	4–6 h [106]
Hydralazine (vasodilator; peripheral smooth-muscle relaxant) [44]	10–25 mg at bedtime	3–7 h [102]
Losartan (angiotensin II receptor antagonist, acting on AT1 receptor subtype) [103]	25–50 mg at bedtime	2 h (metabolite 6–9 h)^a^ [99]
Nifedipine (calcium channel blocker)^b^ [104]	30 mg at bedtime	2 h^c^ [105]
Nitroglycerin patch (vasodilator) [44]	0.1 mg/h patch at bedtime (remove patch in morning)	3 min [100]

*AT1* angiotensin type 1 receptor

^a^Terminal half-life

^b^Prolonged duration of hypotensive effect may worsen orthostatic hypotension in the morning [104]

^c^Elimination half-life of immediate-release formulation

### Other Considerations for Treatment and Future Directions

Treatment of patients with coexisting nOH and nSH can be medically complex, and management may often require collaboration among neurology, cardiology, and other specialties with expertise in the management of autonomic disorders, especially if treatment of nSH worsens the symptoms of nOH.

Currently, much of the data on the management of coexisting nOH and nSH are from patient cohorts with a variety of autonomic failure disorders. An important area for future research may be further understanding of how to individualize treatment for both nOH and nSH based on specific underlying pathophysiology (e.g., by underlying diagnosis of central or peripheral autonomic dysfunction) and level of sympathetic activity [107]. Although some studies have indicated differential treatment effects on BP when patients are grouped by underlying diagnosis (i.e., central/preganglionic or peripheral/postganglionic ANS dysfunction) [24, 108, 109], or biomarkers thereof (eg, supine plasma norepinephrine levels as a marker of peripheral sympathetic denervation) [110], clear evidence for tailoring the management of nOH or nSH by underlying pathophysiology has not yet been identified. Additional areas for future research include a better understanding of the morbidity associated with BP dysregulation in autonomic failure populations and the potential benefits of effective simultaneous management of coexisting nOH and nSH.

## Conclusions

In patients with autonomic failure, cardiovascular autonomic dysfunction frequently occurs and can manifest as coexistent nOH and nSH. Because of opposing hemodynamics, the co-management of these two opposing conditions can present a clinical challenge; however, balanced management is necessary to alleviate symptoms, improve quality of life, and mitigate associated short- and long-term risks (e.g., syncope, falls from fainting, cardiovascular complications). When treating patients with symptomatic nOH and coexisting nSH, clinicians should individualize treatment by considering the presence of nSH and the risk of increased supine BP associated with agents used to treat nOH and managing nSH with nonpharmacologic measures initially. If required, prudent use of short-acting antihypertensives can be used at night to manage nSH.
